# Toward the Discovery of Vaccine Adjuvants: Coupling *In Silico* Screening and *In Vitro* Analysis of Antagonist Binding to Human and Mouse CCR4 Receptors

**DOI:** 10.1371/journal.pone.0008084

**Published:** 2009-11-30

**Authors:** Matthew N. Davies, Jagadeesh Bayry, Elma Z. Tchilian, Janakiraman Vani, Melkote S. Shaila, Emily K. Forbes, Simon J. Draper, Peter C. L. Beverley, David F. Tough, Darren R. Flower

**Affiliations:** 1 The Jenner Institute, University of Oxford, Newbury, Berkshire, United Kingdom; 2 Institut National de la Santé et de la Recherche Médicale Unité 872, Centre de Recherche des Cordeliers, Université Pierre et Marie Curie - Unité Mixte de Recherche en Santé 872/Université Paris Descartes - Unité Mixte de Recherche en Santé 872, Paris, France; 3 The Peter Medawar Building for Pathogen Research, University of Oxford, Oxford, Oxfordshire, United Kingdom; 4 Department of Microbiology and Cell Biology, Indian Institute of Science, Bangalore, India; 5 The Jenner Institute, University of Oxford, Oxford, Oxfordshire, United Kingdom; Sabin Vaccine Institute, United States of America

## Abstract

**Background:**

Adjuvants enhance or modify an immune response that is made to an antigen. An antagonist of the chemokine CCR4 receptor can display adjuvant-like properties by diminishing the ability of CD4+CD25+ regulatory T cells (Tregs) to down-regulate immune responses.

**Methodology:**

Here, we have used protein modelling to create a plausible chemokine receptor model with the aim of using virtual screening to identify potential small molecule chemokine antagonists. A combination of homology modelling and molecular docking was used to create a model of the CCR4 receptor in order to investigate potential lead compounds that display antagonistic properties. Three-dimensional structure-based virtual screening of the CCR4 receptor identified 116 small molecules that were calculated to have a high affinity for the receptor; these were tested experimentally for CCR4 antagonism. Fifteen of these small molecules were shown to inhibit specifically CCR4-mediated cell migration, including that of CCR4^+^ Tregs.

**Significance:**

Our CCR4 antagonists act as adjuvants augmenting human T cell proliferation in an *in vitro* immune response model and compound SP50 increases T cell and antibody responses *in vivo* when combined with vaccine antigens of *Mycobacterium tuberculosis* and *Plasmodium yoelii* in mice.

## Introduction

Adjuvants are substances added to vaccines to enhance or modify the concomitant immune response and induce protection. Virtually all current human subunit vaccines incorporate adjuvants in addition to pathogen-derived antigenic molecules. The use of adjuvants has two main benefits. First, the increased immune response provides better and longer lasting protection against the pathogen and second, the use of an adjuvant allows the dose and dosing regime of the antigen(s) to be decreased and modulated, reducing the cost and logistical complexity of administering vaccines. The principal adjuvants licensed for human use are alum salts and oil-in-water emulsions.

Adjuvants work via many mechanisms and take many forms. Many adjuvants act by stimulating pattern recognition receptors (PRRs) present on cells of the innate immune system, which is the primary bulwark against invading pathogens. PRRs have been found to recognize pathogen associated molecular patterns (PAMPs), which are molecules present in pathogens such as bacterial lippolysaccharides or viral DNA or RNA that differ from mammalian molecules and are thus seen as foreign [1]. Apart from having an immediate function as the first line of defense, the innate immune system also triggers adaptive cellular and humoral immune responses. These provide immunological memory so that the response is greater when the antigen or pathogen is re-encountered. Development of robust protective immunological memory is the central aim of vaccination. In the era of modern vaccinology, adjuvants should have well-defined molecular targets, interacting with specific receptors on cells that have capacity to modulate the course, quality and intensity of the immune response. For receptors that exacerbate or initiate the immune response, such as Toll-like receptors, we need to find adjuvants with agonistic properties. Alternatively, for inhibitory or regulatory receptors, then we need antagonists able to abrogate the suppressive effect of cellular populations with inhibitory or regulatory characteristics.

Receptor-targeted small molecule adjuvants (SMA) are among the most under-explored types of immunomodulatory adjuvants. Examples include: imidazoquinolines (Imiquimod and Resiquimod), which target Toll-like receptors (TLRs), specifically TLR-7 and-8, and were developed as nucleoside analogues for anti-viral or anti-tumour therapy; Bestatin (a tumour adjuvant acting as an inhibitor of aminopeptidase N [CD13]); Levamisole and Bupivacaine (both DNA vaccine adjuvants). Other examples of non-macromolecular adjuvants include monophosphoryl-lipid A, muramyl dipeptide, QS21, PLG, Seppic ISA-51 and CpG oligonucleotides. Optimised CpG oligonucleotides, which target TLR-9, are now entering late phase trials as adjuvants for the poorly immunogenic Hepatitis B vaccine.

Hitherto, the search for novel adjuvants has by no means been a systematic process. The number of potential targets is large and the variety of adjuvants–macromolecules, natural products, small molecules, and combinations thereof–has precluded such a strategy. Focusing on SMAs targeting chemokine receptors, we propose the use of virtual screening as a means of greatly accelerating the process of adjuvant discovery in either an academic or a commercial setting.

Three-dimensional virtual screening, whereby a large number of small molecules are docked into the three-dimensional model of a protein receptor, is an important tool in the field of drug discovery and optimisation. The identification of potential lead compounds from databases of small molecules significantly reduces the time spent on experimental screening and is therefore now an integral part of drug design. There is particular interest in developing drugs which are agonists or antagonists of G-protein coupled receptors (GPCR), a superfamily of transmembrane proteins responsible for the transduction of a variety of extracellular signals into an intracellular response [2], [3].

Chemokine receptors are a family of GPCRs that transduce signals from chemokines, leukocyte chemoattractant peptides secreted by several different cell types both constitutively and in response to inflammatory stimuli [4], [5]. Chemokines can be divided into 4 families based on the arrangement of highly conserved cysteine residues in the amino terminus of the protein. The largest families are the CC and CXC families; the former contains a characteristic motif of two adjacent cysteine residues within the protein sequence while in the latter they are separated by a single amino acid. Chemokines and their receptors play a pivotal role in numerous biological processes, including immune homeostasis, inflammation, angiogenesis, hematopoiesis, brain and heart development.

Chemokine receptors are viable targets for adjuvant discovery. CCR4, which serves as the receptor for two chemokines (CCL17 and CCL22) [6], is of particular interest because it is expressed by regulatory T cells (Tregs), a subset of T cells which normally functions in the downregulation of immune responses [7]–[9]. While elucidating all the diverse mechanisms by which Tregs inhibit immune responses remains a subject of active investigation, a principal means in which they function is through interaction with antigen-presenting dendritic cells (DC) [10]–[13].

Tregs maintain DC in an immature state, in which they are poor stimulators of T cell responses. Since CCR4-mediated migration in response to DC-secreted chemokines is crucially involved in DC-T cell interactions [9], [14]–[16], CCR4 antagonism could enhance immune responses by interfering with the inhibitory function of Tregs. In addition, CCR4 antagonists may possess the ability to alter the type of immune response generated, based on the differential expression of CCR4 on T cell effector subsets. In particular, CCR4 is expressed on Th2-type CD4^+^ T cells which have been linked to allergic inflammatory diseases such as asthma, atopic dermatitis and allergic rhinitis, but is not expressed by Th1 T cells that typically are involved in cell-mediated protection against infection. In keeping with a role for CCR4 on Th2 cells, anti-CCL17 and anti-CCL22 antibodies have both been observed to have efficacy in murine asthma models. Given that Th1 and Th2 responses are mutually antagonistic, CCR4 antagonists may act as adjuvants that direct the immune response towards a Th1-type response.

Chemokines and other large peptide ligands bind the extracellular loop scaffold, a combination of the extracellular loops and N terminus of the receptor, and thus only partially penetrate the transmembrane core [17]. Small molecule agonists and antagonists of the molecule do not interact with the extracellular loops but instead occupy a cavity within the transmembrane region of the receptor that corresponds to a typical ligand-binding site. Many commercially successful compounds act as GPCR ligands and display several commonalities: the biphenyl tetrazole moiety is, for example, a common motif. However, the lack of sequence homology between GPCR subgroups means that no generalisations can be assumed.

**Figure 1:**
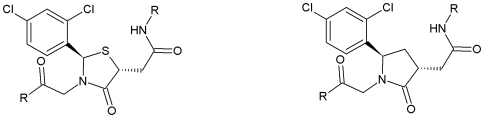
Structure of the thiazoline (a) and lactam (b) derivatives.

Several CCR4 small molecule antagonists have previously been developed, primarily with the aim of reducing T cell migration to sites of inflammation. These antagonists have primarily been developed based upon heterocylic rings. Allen *et al.* [18] produced a series of thiazolidinone derivatives that all take the form of three components extending from a central ring, a general structure or pseudo-pharmacophore similar to many previously identified chemokine receptor antagonists (see Figure 1a). The components take the form of a left side tethered amide, a right side amide and a central aromatic ring. Optimisation of these three components generated a series of inhibitors with a potency range of 100–200 nM. Despite showing a good affinity for the CCR4 receptor, the thiazolidinones showed poor *in vivo* absorption. Subsequently, the thiazolidinone core was replaced by a lactam (see Figure 1b) [19]. The replacement of a sulphur atom by a carbon removed from the compounds a potential centre for oxidative metabolism. Although the lactams were more efficiently absorbed and possessed enhanced chemotaxic antagonism, they also had reduced potency.

**Figure 2:**
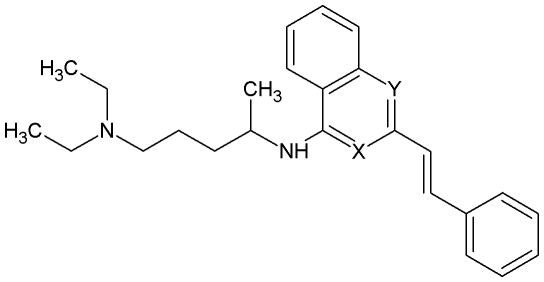
Structure of the quinazoline, quinoline and isoquinoline derivatives where either X or Y or both equal N.

Separately, a series of closely related quinazoline, quinoline and isoquinoline derivatives (see Figure 2) were identified which were also CCR4 receptor antagonists. The compounds inhibited binding of radiolabelled CCL22 and CCL17 to CCR4 receptors on the surface of CEM cells and also inhibited *in vitro* migration of the cells in response to CCL17. Structural modification was undertaken to separate CCR4 antagonism from the cytotoxicity of the compounds by varying the central aromatic core and terminal aromatic moiety. Subsequent optimisation trials significantly reduced cytotoxicity while retaining antagonism.

Previously, structure-based virtual screening using homology models of GPCRs has demonstrated its validity [20] by constructing 3D models of three GPCR proteins (the Dopamine D3, Muscarinic M1 and Vasopressin V1a receptors) and testing their ability to identify molecules with agonistic and antagonistic properties. Further work on 5-HT agonists demonstrated that the most efficient method of library design for a GPCR screening utilizes a combination of a ligand-based similarity search and structure-based screening to identify molecules with binding properties [21]. Amongst other studies, a virtual screening protocol based on a GPCR model was used to identify Cannabinoid bioactive antagonists [22].

Here, we use a combination of fuzzy ligand similarity, homology modelling of the CCR4 chemokine receptor, and structure-based virtual screening to identify affine small molecule ligands of CCR4. Experimental validation *in vitro* confirms that these molecules have antagonistic properties and are capable of inhibiting the activity of Tregs. Further, *in vivo* validation is consistent with these molecules acting as adjuvants.

## Results

### Homology Modelling

**Figure 3:**
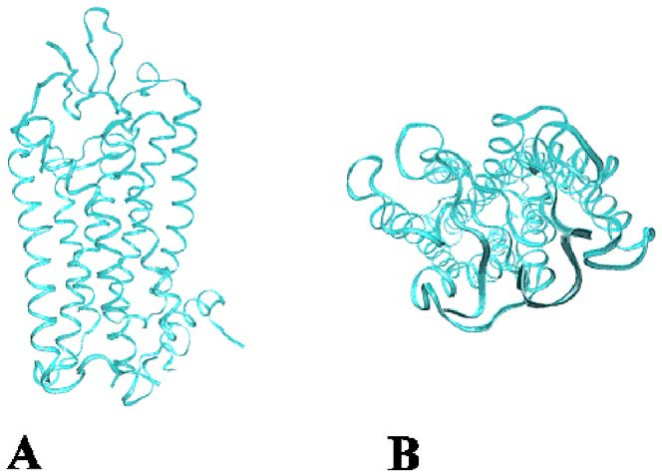
The crystal structure of bovine rhodopsin.

In the absence of an experimentally determined structure for the CCR4 chemokine receptor, it was necessary to create a homology model from related proteins with determined structures. The first high resolution structure of a GPCR, bovine rhodopsin, was published in 2000 by Palczewski *et al.* (see Figure 3a–b) [23]. Although there was strong evidence that all GPCRs had the same overall structure, this was not confirmed until the publication of a second GPCR structure, β2 adrenergic receptor (see Discussion) [24]. A comparison of these two structures shows that the transmembrane region is conserved but that there is a significant difference in the extracellular and intracellular regions [25], [26]. The initial parts of this study - model building and virtual screening - were conducted before the β2 adrenergic receptor became available; thus bovine rhodopsin was used as the template for CCR4.

Homology modeling is a four-step process by which homologues are first identified and then form the structural template, the target sequence is aligned to the template, a model is built with this sequence and optimised, and, finally, the model is evaluated [27]. The structure of CCR4, as is the case for all GPCRs, comprises seven α-helices in a flattened two-layer structure joined by three intracellular and three extracellular loops. The transmembrane region of the molecule is composed of seven highly conserved segments of 20–30 consecutive residues with a high degree of hydrophobicity. The transmembrane sequences were predicted using a transmembrane prediction algorithm (see Table 1) and docked together using the bovine rhodopsin structure as a scaffold.

**Table 1:** The predicted transmembrane (TM), intracellular loop (IL) and extracellular loop (EL) regions of the human CCR4 protein.

N terminus	mnptdiadttldesiysnyylyesipkpctkegi
TM1	kafgelflpplyslvfvfgllgnsvvvlvlfky
IL1	Klrs
TM2	**mtdvyllnlaisdllfvfslpfwgyyaadq**
EL2	Wfg
TM3	lglckmiswmylvgfysgiffvmlmsidrylaiv
IL2	havfslrart
TM4	ltygvitslatwsvavfaslpgfl
EL2	fstcyternhtycktkyslnsttw
TM5	kvlssleinilglviplgimlfcysmiirt
IL3	lqhcknekknk
TM6	avkmifavvvlflgfwtpynivlfletlve
IL3	levlqdctferyldyaiq
TM7	atetlafvhcclnpiiyfflgekfr
C terminus	kyilqlfktcrglfvlcqycgllqiysadtpsssytqstmdhdlhdal

**Figure 4:**
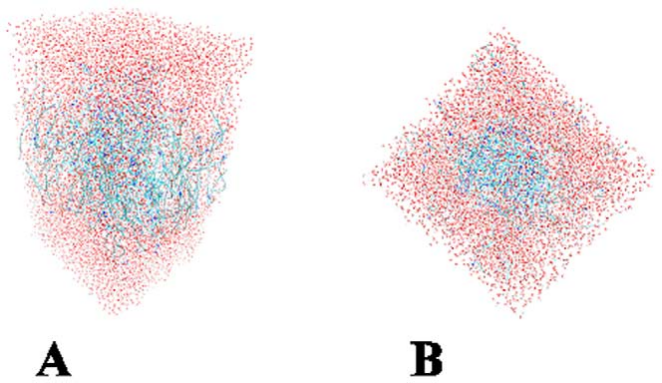
The CCR4 homology model inserted into a LMP and fully solvated. The water atoms (red) form a box around the protein and DPP atoms (blue).

Hydrophobic profiles, derived from GPCR multiple sequence alignments, were used to assign helical transmembrane regions. The extracellular and intracellular loops as well as the termini of the molecule were harder to model due to the low homology between CCR4 and bovine rhodopsin in this region, as well as the limitations of loop modelling methods. Extended loop conformations have highly variable conformations and are particularly difficult to model. The termini and loops were therefore added in an extended conformation. Homology models of both the human and mouse CCR4 structures were generated. Completed CCR4 structures were then inserted into optimised lipid bilayers [28], [29] and bad contacts between the protein and lipid were eliminated. The protein-ligand complexes were fully solvated and an energy minimisation simulation was used to optimise the protein structures (see Figure 4a–b).

### Virtual Screening

At its most general, a pharmacophore summarizes structural information common to ligands exhibiting a particular activity. Based upon previously determined antagonists for chemokines, we devised a set of screens that mimicked the behaviour of a low-level pharmacophore: it specified that molecules should have molecular weight >500, contain at least two 5- or 6- membered aromatic rings, and at least one nitrogen atom. The CHEMBRIDGE, TIMTEC and SPECS databases were scanned using this pharmacophore and 13,000 compounds were thus selected for evaluation by docking. The ligands were evaluated using the GOLD docking program [30] using the Goldscore fitness function.

Previously, residues within the transmembrane region that are involved in the binding region of human CCR4 have been identified by mutational analysis; they include: Leu 42 and Tyr 46 (TM1), Trp 45 (TM2), Ile 113, Tyr 117 and Phe 121 (TM3), Ser 203 and Ile 206 (TM5), Tyr 258, Asn 259 and Leu 262 (TM6) and Glu 290 (TM7). The residues implicated in the receptor-ligand binding identified a cavity within the transmembrane region of the CCR4 receptor corresponding to a typical ligand-binding site [31]. The cavity forms the shape of a distended teardrop with a size and relative disposition defined by relative positions of the seven transmembrane helices.

Two protein models were generated using homology modeling: human CCR4 and mouse CCR4. Manual docking of known chemokine ligands was consistent with a common mode of antagonist binding for both receptors. For both human and mouse receptors, GOLD was directed to dock each ligand within the cavity by specifying that the docking must occur within 4 Å of at least one the specified residues. Versus the human receptor, the highest ranked molecule had a high Goldscore value of 71.03. For logistic reasons, the top 116 were selected based on the Goldscore versus human CCR4. The lowest ranked compound had a Goldscore of 54.69. The 116 molecules were tested *in vitro* for CCR4 receptor antagonism. For these compounds, scores versus mouse CCR4 were decreased by 10; overall, mouse and human Goldscores had a correlation coefficient of 0.49 (data not shown).

Fifteen of the small molecules were shown to inhibit significantly *in vitro* CCR4-mediated migration at low concentrations, mediating 50% inhibition of migration at low nanomolar level or below (see Table 2). The docked structures were minimized again in order to improve their orientation within the groove. The small molecules did not have standard parameters so they were calculated using the program *antechamber* [32]. The residues that contact each of the small molecules are shown in Table 3.

**Table 2:** Inhibition data from 16 small molecules selected by virtual screening. Antagonists shown to inhibit Treg migration shown in bold.

Compound	MW	Concentrations required to inhibit 50% of CCRF-CEM cell migration in the chemotaxis assay (M)
CB4	534.1135	1.80×10^−09^
CB16	506.3258	2.49×10^−11^
CB20	599.8731	5.11×10^−10^
**CB28**	**548.6657**	**3.13×10^−10^**
SP20	501.3615	1.14×10^−11^
SP27	598.0112	2.75×10^−10^
SP30	525.4454	5.90×10^−11^
SP32	561.4423	3.33×10^−11^
SP35	536.4247	3.55×10^−12^
**SP40**	**571.4658**	**7.90×10^−12^**
**SP45**	**628.8046**	**5.14×10^−12^**
**SP46**	**565.9365**	**2.34×10^−12^**
SP48	617.5108	1.76×10^−11^
**SP50**	**531.4835**	**9.78×10^−11^**
**TT3**	**636.441**	**8.59×10^−12^**

**Table 3:** Contact residues for the eight selected small molecules. All residues contain are within 4 Å of a ligand atom. Residues in bold are common to all six ligands.

Residue	Transmembrane	TT3	SP50	SP46	SP40	SP45	CB28
Leu 92	TM2						
Tyr 117	TM3		X	X	X	X	
Phe 121	TM3	X	X	X	X	X	
Tyr 122	TM3	X			X	X	
Phe 126	TM3	X					
Phe 167	TM4	X					
Leu 201	TM5	X					
Ser 202	TM5						X
Ser 203	TM5						X
Leu 204	TM5						X
Glu 205	TM5			X		X	X
**Ile 206**	TM5	**X**	**X**	**X**	**X**	**X**	**X**
Asn 207	TM5						X
Leu 209	TM5			X	X		
Gly 210	TM5	X					
Trp 255	TM6		X	X	X	X	
Pro 257	TM6		X	X	X	X	X
**Tyr 258**	TM6	**X**	**X**	**X**	**X**	**X**	**X**
Asn 259	TM6	X					X
Ile 260	TM6						X
Val 261	TM6		X	X		X	X
Leu 262	TM6	X	X	X	X	X	X
Phe 263	TM6						X
Thr 266	TM6						X
Ala 285	EL3		X			X	
Ile 286	EL3		X	X	X		
Gln 287	EL3		X	X	X	X	X
Ala 288	TM7						X
Thr 289	TM7						X
**Glu 290**	TM7	**X**	**X**	**X**	**X**	**X**	**X**
Thr 291	TM7					X	
Ala 293	TM7					X	X
Phe 294	TM7		X	X	X	X	
Val 295	TM7		X	X	X	X	

### In Vitro Validation

**Figure 5:**
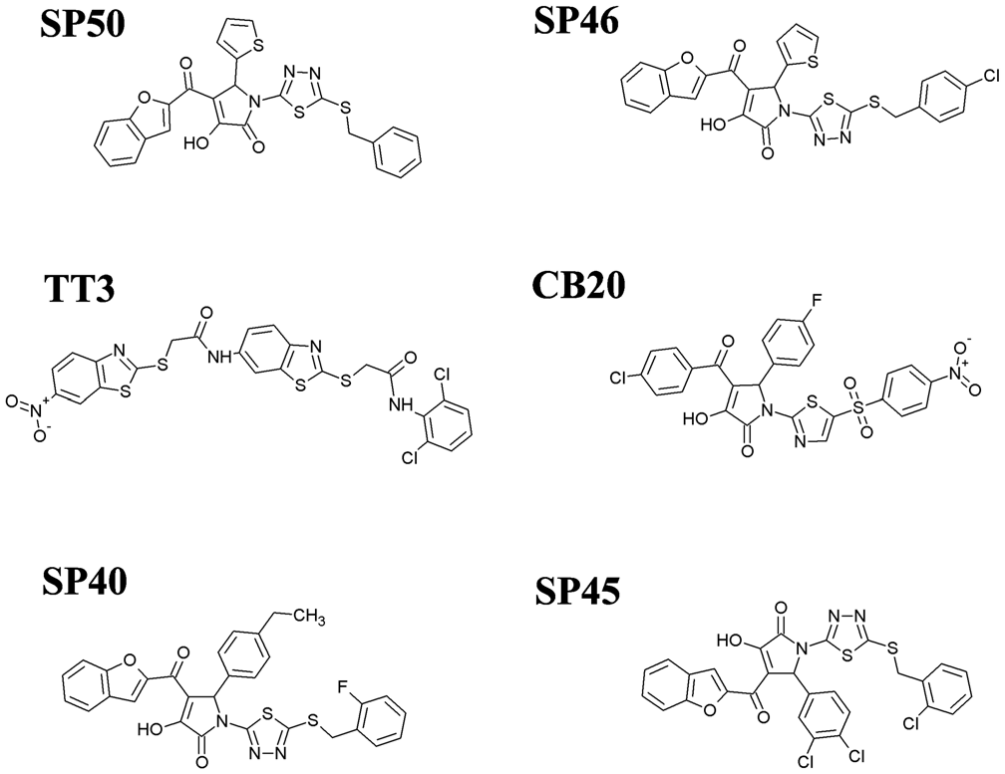
The conventional 2D representation of the six selected ligands showing inhibitory properties for CCR4-mediated migration.

**Figure 6:**
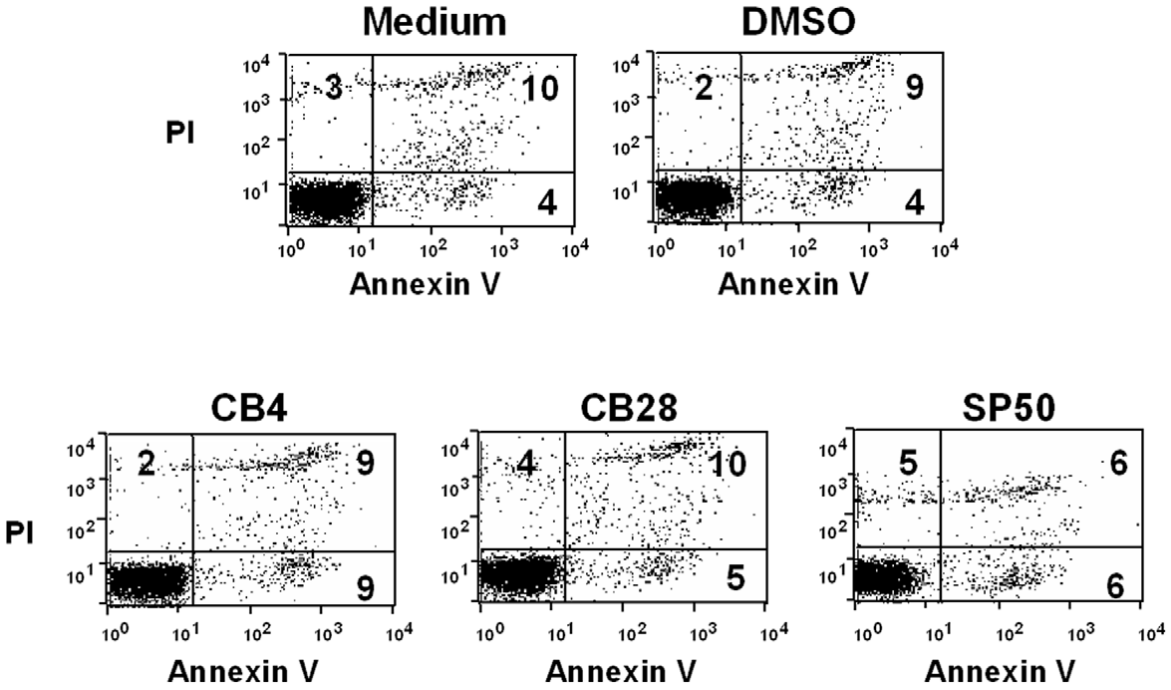
Assessment of CCRF-CEM cell viability by propidium iodide (PI) and annexin V labeling following treatment with CCR4 antagonists. Propidium iodide (PI) labels cells lacking intact plasma membranes and hence at an advanced stage of cell death. Annexin V labels cells at early and late stages of apoptosis. Plots show representative data for cells cultured in medium alone or in medium to which solvent (DMSO) or antagonist has been added. Numbers indicate percentage of cells in each quadrant.

Upon receiving maturation and activation associated signaling, DC secrete CCL22 and CCL17, the ligands for CCR4 [33]. The binding of these ligands to CCR4 helps to guide Tregs towards DC, mediates contact between these two cell types and thus inhibits activation of DC and the DC-mediated T cell response. If CCR4^+^ Tregs fail to effectively compete with naive T cells for access to DC because CCL22 and CCL17-binding to CCR4 is blocked, this should result in firm contact between T-DC and increased activation and differentiation of vaccine antigen-specific effector T cells. As reported elsewhere [34], 15 of the 116 top ranked molecules from virtual screening specifically inhibited CCL22-mediated chemotaxis of a CCR4^+^ human Caucasian acute T lymphoblastoid leukaemia cell line CCRF-CEM, yet had no effect on migration mediated through CXCR4, which is also expressed on CCRF-CEM cells, demonstrating their selectivity for CCR4. Importantly, 6 molecules (SP45, SP50, CB28, TT3, SP46, SP40) were shown directly to block CCR4-mediated migration of human Tregs in response to CCL17 and CCL22. The structures of these 6 small molecules are shown in Figure 5. All 6 antagonists inhibited significantly CCL22-mediated Treg migration and up to 49.8% inhibition was observed when Tregs from six individual donors were examined, whereas none of the 6 compounds affected cell viability (Figure 6). In addition, all of the molecules inhibited Treg migration in response to another CCR4 ligand, CCL17 (up to 57.2% inhibition of migration) as well as CCL22- and CCL17-mediated migration of human Th2 cells. The CCR4 antagonists enhanced significantly DC-mediated human T cell proliferation in an *in vitro* model of immune response when Tregs were present in the CD4^+^ T cell pool, consistent with the hypothesis that molecules that inhibit Treg migration should possess adjuvant activity.

### In Vivo Validation

**Figure 7:**
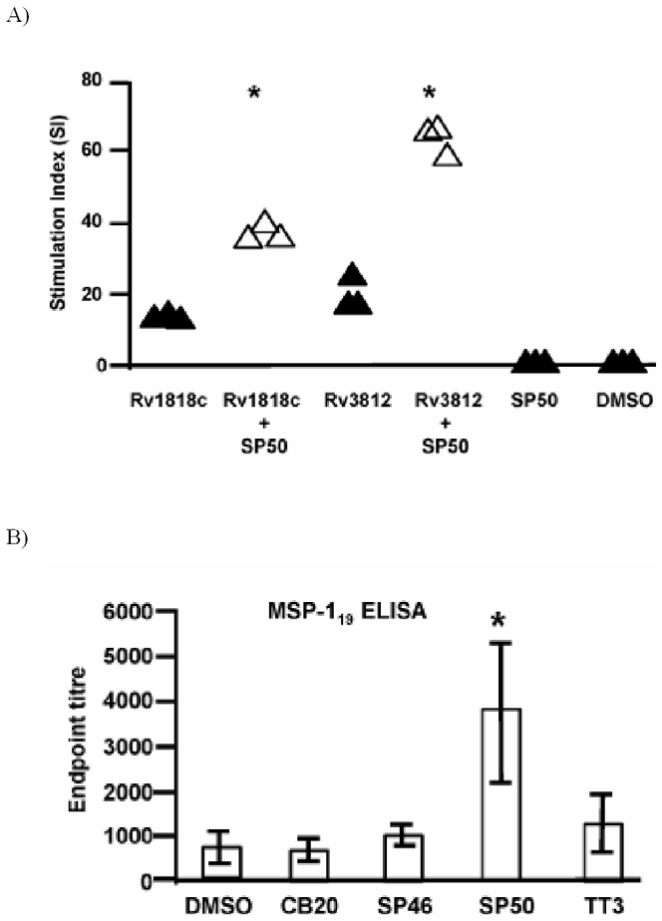
CCR4 antagonists enhance cellular and humoral responses in mice. (*a*) Effect of SP50 on T cell priming to *Mycobacterium tuberculosis* protein antigens Rv1818c or Rv3812. Splenocytes (0.5×10^6^) from mice immunised with the indicated proteins+SP50 or DMSO were cultured *in vitro* in the presence of 5 µg/ml of either Rv1818c or Rv3812 recombinant proteins for 5 days. Cells from three mice per group were tested individually in quadruplicate wells. Antigen-specific T cell proliferation was measured by thymidine incorporation assay and data are presented as stimulation index (SI, mean cpm of the antigen stimulated wells/mean cpm of control wells). Significant differences are indicated by asterisks * p<0.05. The results are from one experiment. (*b*) IgG serum antibody responses against MSP-1_19_ measured by ELISA 14 days post vaccination with Ad-MSP-1_42_ plus the indicated compounds or DMSO control. Three mice per group were tested individually. Columns represent the mean±SD. Significant differences are indicated by asterisks * p<0.05. Similar results were obtained in two independent experiments.

Prior to testing the adjuvant activity of CCR4 antagonists *in vivo*, we first studied the ability of antagonists to inhibit the CCL22-mediated migration of the murine CCR4^+^ T cell hybridoma B9.1. These migratory assays were undertaken using a similar protocol to that used for human cells. Four of the 15 compounds (CB20, SP46, SP50 and TT3) were found to inhibit the migration of B9.1 cells up to 55%. We then examined the influence of one of these compounds (SP50) on the immune response to vaccination in mice. The impact of CCR4 antagonists on the cellular immune response was investigated using a CMV vector expressing Rv1818c (CMV1818c) or Rv3812 (CMV3812) proteins from *Mycobacterium tuberculosis* [35]. Simultaneous administration of SP50 with CMV1818c or CMV3812 enhanced significantly the Rv1818c and Rv3812-specific CD4^+^ T cell proliferative immune response (Figure 7a). Interestingly, no significant changes were observed in the percentage of Tregs in the spleen of mice injected with antagonist alone (3.75–4% CD4^+^FoxP3^+^ T cells in SP50 injected mice versus 4–4.5% CD4^+^FoxP3^+^ T cells in control groups), thus confirming that CCR4 antagonists *per se* do not modify the population of Tregs.

The potential for CCR4 antagonists to enhance antibody responses was examined using Human Adenovirus 5 encoding the 42 kD region of the merozoite surface protein-1 (Ad-MSP-1_42_) from *Plasmodium yoelii*. Ad-MSP-1_42_ induces a strong antibody response after two weeks to the 19 kD fragment of MSP-1 (MSP-1_19_), which is thought to mediate protective immunity [36], [37]. Notably, despite the considerable intrinsic immunogenicity of this vaccine, the titre of MSP-1_19_-specific IgG was significantly increased by co-injection of SP50 (Figure 7b). Although the three other compounds tested had no effect, we did not carry out thorough dose response experiments nor did we investigate the timing of adjuvant relative to antigen administration. However SP46 and TT3 increase cellular immune responses to mycobacterial antigens as previously reported suggesting that these antagonists selectively potentiate cellular immunity [34].

## Discussion

Kornbluth and Stone have recently hailed a new golden age of vaccine discovery focusing on the exploitation of adjuvants as immunomodulators able to enhance immunogencity of subunit and peptide-based vaccines [38]. They group adjuvants into stimulatory and suppressive immunomodulators. Immunostimulatory adjuvants include Toll receptor agonists; agonists of CD40 and other members of the TNF receptor superfamily such as OX40L, 4-1BBL, CD30L, LIGHT, CD27L/CD70, and GITRL; and agonists of the Nod-like Receptor system. Adjuvants that function by modulating immunosuppressive arms of the immune system include neutralizing antibodies to anti-inflammatory cytokines or antagonists to molecules such as CTLA-4 that provide negative signaling to innate immune cells. Our molecules also fall into this second category.

Compared to biogenic macromolecules or synthetically intractable natural products, small molecule adjuvants have many potential advantages. As small drug-like molecules, it may be possible to tailor their properties precisely, using the standard pharmaceutical toolkit - structure- and ligand-based virtual screening, parallel synthesis, and medicinal chemistry - properly to explore their specificity and properties. It should also be possible to develop an understanding of how to manipulate rationally the structure of these compounds so as to generate molecules with improved *in vivo* characteristics. While a drug with a once-a-lifetime or once-a-year dosing does not require all the properties that might be expected in a modern drug, altering them to be as close as possible to optimum pharmacokinetic properties would be advantageous.

Here we have used virtual screening to identify CCR4 antagonists which act as adjuvants for both cellular and humoral immune responses. Effective three-dimensional virtual screening is reliant upon an accurate model of the receptor. There are very few experimentally-determined high-resolution structures of membrane proteins available in the Protein DataBank due to difficulties in their over-expression, purification and crystallization [39]. Where the similarity between sequences is less than 30%, it can be extremely difficult to align sequences in a certain and unambiguous way; this region of similarity is often described as the ‘twilight zone’. The lower the sequence similarity between the target and template protein, the more difficult it becomes to undertake a successful alignment between them. Despite the low sequence homology (22.7%) between bovine rhodopsin and the chemokine receptors, it was possible to identify the transmembrane regions within the chemokine sequence and build them up as idealised α-helices. This is because all GPCRs share a common pattern of hydrophobicity and similarity of structure even when there is sequence divergence; thus a reasonable model of the transmembrane region of the chemokine receptor can be generated. The validity of using homology modelling to generate GPCR structures is supported by comparison with the β2-adrenergic receptor. It is very similar in terms of the relative orientation of the seven transmembrane helices, suggesting a degree of universal conformity for the transmembrane region of GPCRs. The conformation of the second extracellular loop, which often constitutes the top of the ligand binding site, is, however, quite different in the two structures.

**Figure 8:**
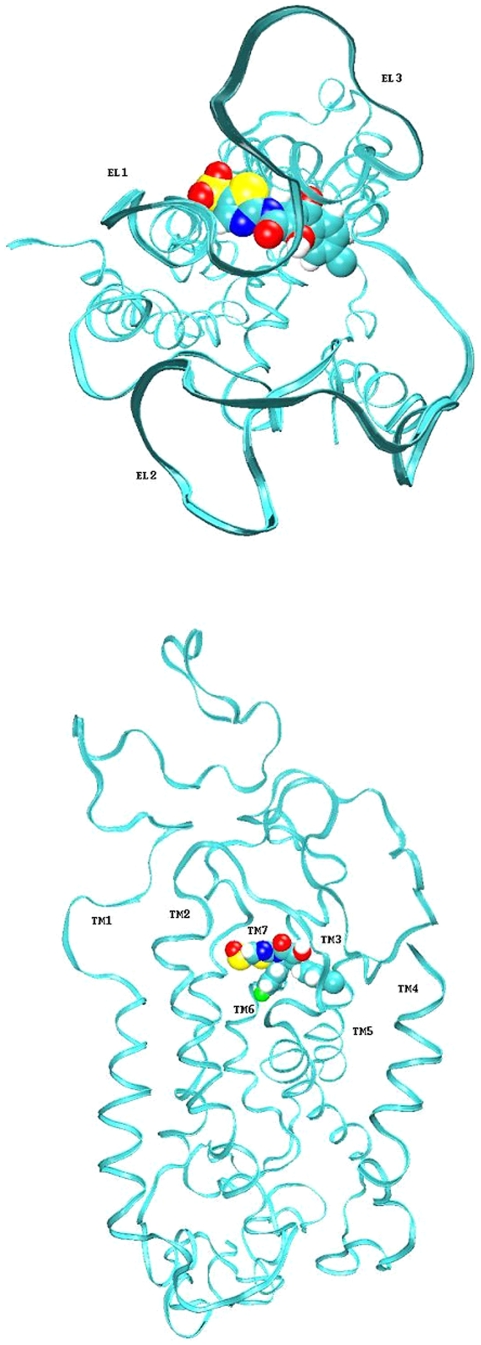
CB20 ligand docked into the CCR4 homology model.

Attempts to develop small molecules antagonists for chemokines have focused on the CCR5 and CXCR4 receptors because an antagonist might have therapeutic potential in the inhibition of HIV-1 virus uptake [3]. Docking studies using the antagonist TAK-779 [40] and a model of the related CCR5 receptor identified a region of the binding pocket that may act as a conserved binding point for chemokine antagonists sharing an ammonium group [41]. The benzyl-pyron-ammonium-group of TAK-779 interacts with transmembrane helices 1, 2 and 7. Selectivity of TAK-779 was analysed using site-directed mutagenesis and is in agreement with observed structure-activity relationships. The binding is dependent on the ammonium groups being in close contact with the conserved CCR5 residue Glu 283 (Glu 290 in CCR4). The introduction of a single point mutation into a functionally active receptor can lead to a decrease in binding affinity, indicating the residue is vital to the ligand-receptor interaction. It is likely that high affinity receptor-ligand binding is partly dependent on strong interactions with a few key residues. There are also non-conserved residues within transmembrane regions TM3, TM5 and TM6 that can impair receptor selectivity. Previous attempts to model the ligand interactions of the CCR5 ligand identified the same cavity within the chemokine structure but showed the binding of the ligand to be primarily dependent on interaction with the TM1, TM2, TM3 and TM7 helices. The docked structure of CB20 in the human CCR4 receptor homology model is shown in Figure 8a–b. The docked models indicate that the ligands maintain key interactions with the TM7 but not the TM1 and TM2 helices. Residues Ile 206, Tyr 258 and Glu 290 (on the TM5, TM6 and TM7 helices respectively) seem to be fundamental to the interaction, in particular, Glu 290; this interacts with positively charged moieties on the bound ligand. The other end of the molecule is predominantly hydrophobic and interacts with Ile 206. The molecules are all linear and stretch across the length of the groove to interact with both the TM5 and TM7 helices. There is also a slight interaction with extracellular loop III (285–287). It is possible that a less linear molecule, such as TAK-799 that binds the CCR5 receptor, would not position itself directly across the groove. It is also possible that the parameters defined by *antechamber* do not allow our small molecules sufficient flexibility to optimize their position within the cavity [32]. The human CCR4 model was subsequently modified to produce a mouse CCR4 receptor model. There is strong homology between the two structures and the extended tear drop structure of the active site is maintained.

When tested *in vivo* with vaccines in mice, enhanced immunogenicity was observed with the CCR4 antagonist SP50 acting as an adjuvant for both cellular and humoral immune responses. The lack of effects on the humoral response of the three other compounds tested (CB20, SP46 and TT3) might be because an inappropriate dose or time of administration was used, although SP46 and TT3 increase cellular immune responses to mycobacterial antigens when used in combination with Modified Vaccinia Ankara expressing Ag85A from *M. tuberculosis* (MVA85A) [34]. Sp50 has previously been demonstrated to potentiate the humoral response to another antigen, recombinant hepatitis B surface antigen (HBsAg) [34], which is a poorly immunogenic antigen often requiring several immunizations to generate protective antibody titers in humans. Strikingly in the present experiments, in addition to its effect on weakly immunogenic HBsAg, SP50 potentiates cellular and humoral responses to Mycobacterial antigens and a malaria antigen administered in viral vectors that have strong inherent adjuvanticity. We suggest that this augmentation may be because these viral vectors mediate adjuvant effects by ligating pattern recognition receptors while SP50 interferes with regulatory cell function.

If CCR4+ Tregs fail to compete effectively with naive T cells for access to DC because CCL22 and CCL17-binding to CCR4 is blocked then this should result in the reduced influence of Tregs on both DC and T cell populations and as a consequence an increased antigen-specific cellular and humoral response. In fact, several experimental models have demonstrated that inhibition of Treg function results in markedly superior immune responses to foreign and tumour antigens, virus infection and vaccines [42]–[44]. However, these experiments were typically performed by using therapeutic monoclonal antibodies either to deplete Tregs or to block CD25, an IL-2 receptor α-chain expressed by Tregs.

We believe that CCR4 antagonists may have advantages over these methods of blocking Treg functions. First, antibody-mediated inhibition of Treg function might result in severe adverse effects, since Tregs are required for maintenance of immune tolerance. For example, injection of anti-CD25 antibodies alone or in combination with anti-CTLA-4 antibodies into normal animals is known to induce localized autoimmune disease [45], [46]. Second, as compared to small molecules, therapeutic IgG monoclonal antibodies have a longer half-life (about 3 weeks in humans). Indeed, we did not observe any significant changes in the Treg population in the spleen of mice injected with antagonist alone, thus confirming that CCR4 antagonists do not delete the Treg population. Therefore we suggest that it is likely that CCR4 antagonists perform their adjuvant-like function by transiently inhibiting Treg migration. In addition, our *in vitro* experiments support the proposition that CCR4 antagonists could function similarly in humans. Thus, in a transwell system, exposure of T cells to CCR4 antagonists enhanced their proliferation in response to mature DC. This model may mimic the early events of an immune response, where inhibition of Treg recruitment to DC would lead to an increased antigen-specific T cell response.

Aluminum adjuvants are the only adjuvants licensed for human vaccines in the USA. The immune responses elicited by aluminum adjuvants are not likely to confer protection against diseases such as malaria, tuberculosis, and cancer for which Th1 and MHC class I restricted cytotoxic T lymphocyte (CTL) responses are essential for protection [47]. Therefore, adjuvants that can target specific receptors and elicit protective Th1 and CTL immune responses are desirable. Thus, several novel adjuvants, including molecules that target TLRs and NLRs which potently activate antigen presenting cells and Th1 type immune responses are under consideration [48], [49]. Our *in vivo* results in experimental models demonstrate that small molecule antagonists to CCR4 enhance both cellular and humoral immune responses to vaccine antigens. Thus, in the future they can be considered for use with novel vaccine candidates either alone or in synergistic combination with other adjuvants.

In trying to identify novel agonists and antagonists for a receptor, computational chemistry allows a large number of compounds to be screened rapidly. This can act as a filtering system allowing molecules that have been calculated to have affinity for the receptor to be targeted. Virtual screening explores the chemical diversity of potential ligands and structural constraints imposed by the receptor. GPCR agonists and antagonists tend to be larger molecules than commonly favoured in drug design. Helices 3, 5, 6 and 7 are responsible for the majority of the side chain interactions with the bound ligand. It is likely that the CCR4-ligand interactions depend on strong interactions in a few key residues, such as Ile 206, Tyr 258 and Glu 290, as well as weaker ones with the other residues in the CCR4 cavity. Site-directed mutations could be used to establish those residues which are responsible for interacting with particular functional groups, facilitating an augmented understanding of ligand-receptor structure-activity relationships (SAR). Site directed mutations in the TM 5, 6 and 7 regions would be useful in further determining the nature of the receptor-ligand interaction and help towards optimising CCR4 antagonists. All of the ligands have a molecular weight >500 which contravenes Lipinski's rule-of-five regarding the maximum size of a potential drug candidate [50]. Further work will target reducing the molecular weight of the molecules, as well as maximizing their antagonistic properties.

Three-dimensional virtual screening is now known to be an effective and economical way of identifying potential lead compounds with desired activity [51]. In this study, about 14% of the small molecules identified by virtual screening were shown to have a degree of antagonism, remarkable considering the receptor structure was not an experimental structure but a homology model built from a related structure with a low degree of sequence homology. The inclusion of an explicitly rendered lipid bilayer into the energy minimisation simulation may have helped in optimising the quality of the structure. The identified antagonists have been shown to be both potent and specific for the desired target receptor.

Our results re-emphasize the power of virtual screening, by systematising the discovery of small molecule adjuvants through targeting receptors implicated in the innate immune response or in regulating the adaptive immune response. Using virtual screening, we have identified CCR4 antagonists acting as adjuvants for both cellular and humoral immune responses. When our molecules were tested *in vivo* with vaccines in mice, enhanced immunogenicity was observed with SP50. The enhancing effects observed in these experiments are particularly striking given that the vaccine vectors employed are known to be intrinsically immunogenic [35], [37]. Within vaccinology - as it is within drug design and discovery–structure-based virtual screening is an approach of unprecedented power and scope; only wide deployment is needed for virtual screening to realize its full potential.

## Methods

### Model Building

The transmembrane sequences of human CCR4 protein were docked together using the bovine rhodopsin structure as a scaffold. Attwood developed diagnostic fingerprint for rhodopsin-like GPCRs based on common patterns of conservation in the seven transmembrane regions [52]. WHATIF was used to generate the helical transmembrane sections of human CCR4 [53]. The orientation of the helices is calculated with respect to a lipid environment so that hydrophobic faces are orientated into the membrane phase and hydrophilic faces point into the lumen of the protein. The translational and rotational orientation of each helix in the transmembrane bundle is critical to the nature and conformation of the binding site. Hydrophobic areas of the transmembrane bundle will be orientated such that their peak hydrophobicity lies centrally within the lipid plane. This position corresponds to the so-called lipid midpoint plane (LMP). A model of mouse CCR4 was constructed using the optimised human CCR4 structure as a template.

### Energy Minimisation

Hydrogen atoms were added to the human CCR4 structure and the system was fully solvated using water molecules in the TIP3 model [54] by the AMBER program *leapi* [32]. This created a solvent box with dimensions of approximately 40 Å by 50 Å by 120 Å and approximately 110,000 atoms (See Figure 4a–b). All atoms in the simulation were explicitly represented. Two known conserved disulphide bonds between Cy29–Cys276 and Cys110–Cys187 were built into the structure [55]. The energy of the solvated molecular complex was minimised using the general AMBER force field with a steepest descent method that was continued for 50,000 time steps (one time step–one femtosecond) or until the RMSD had fallen below 0.01 Å between successive time steps. In the first stage of minimisation, the transmembrane region and lipid region were frozen in order to allow the loops to order themselves using the transmembrane scaffold. In the second stage, simulated annealing was carried out on the minimized structure. At this stage, all atoms in the systems were allowed free movement. The system was annealed by raising the temperature of the system from 0 K to 500 K over a period of 40,000 time steps and maintaining that temperature for a further 30,000 time steps. The system was then cooled to 0.2 K over a period of 230,000 time steps before being rested at 1 K for a further 300,000 time steps. The CPU of each individual simulation was approximately 500 hours on a 6-processor R12000 SGI Origin 2000. All minimisation and annealing steps were performed using the *sander* program [32]. The process was repeated for the mouse CCR4 model. The docked small molecules were run under the same conditions and time period as the initial energy minimization. *Antechamber* was used to generate parameters for the small molecules.

### Virtual Screening

A database containing structures from a variety of compound suppliers was constructed within UNITY [56] and screened for potentially reactive and undesirable molecules [57]. The resulting database contained ∼450 K molecules. This was pre-screened using a simple pseudo-pharmacophore derived from properties of known Chemokine antagonists: compounds must have a MW>500 and contain two or more 5 or 6 membered aromatic rings and one or more nitrogen atoms. Thirteen thousand compounds were thus selected, and their 3D structure built using CORINA [58], which were tested for interaction with the two modelled CCR4 structures using the GOLD docking program and the Goldscore fitness function. The ligands were docked within a predicted cavity in the transmembrane region of human and mouse CCR4.

### Cell Lines

The human Caucasian acute T lymphoblastoid leukaemia cell line CCRF-CEM (European cell culture collection) and murine T cell hybridoma B9.1, specific for the immunodominant peptide HEL103–117 [59], were cultured in RPMI 1640 with 10% fetal calf serum. Both cell lines express CCR4 and migrate in response to CCR4 ligands.

### Generation of Dendritic Cells

Peripheral blood mononuclear cells (PBMC) were isolated from buffy coats, purchased from the North London Blood Transfusion Centre, by Ficoll-Hypaque density gradient centrifugation. Ethical approval for use of this material was obtained from the Compton Human Subjects Committee. Monocytes were purified by positive selection using CD14 beads (Miltenyi Biotech, Surrey, UK). For generation of DC, monocytes were cultured for 6 days in the presence of RPMI 1640 supplemented with 10% FCS, 50 U/ml penicillin, 50 µg/ml streptomycin, recombinant human (rh) IL-4 (500 IU/10^6^ cells) (R&D Systems Europe, Abingdon, UK) and rhGM-CSF (1000 IU/10^6^ cells) (Immuno Tools, Friesoythe, Germany).

### Isolation of Human CD4+CD25+ Regulatory T Cells

CD4^+^CD25^+^ Tregs were isolated PBMC using a kit from Miltenyi Biotech. The purity of isolated regulatory T cells was over 95% as assessed by flow cytometry.

### Generation of Th2 Cells

Naïve CD4^+^ CD45RA^+^ T cells were purified from PBMC in a 2-step process using magnetic beads (Miltenyi Biotech). First, untouched CD4^+^ T cells were isolated by negative selection. Second, CD45RO^+^ T cells were depleted using CD45RO beads. The remaining CD4^+^ CD45RA^+^ T cells were added to 24-well tissue culture plates that were pre-coated with 10 µg/ml anti-CD3 and anti-CD28 mAbs (R&D systems Europe). Cells were cultured in RPMI 1640/10% FCS in the presence of 10 µg/ml neutralizing anti-IL-12 and IFN-γ mAbs, 10 ng/ml rhIL-2 and 20 ng/ml rhIL-4 (all from R&D systems). After 3 days, 0.5 ml of 4 ng/ml IL-2 was added to the cultures. At day 6, cells were harvested, washed and the stimulation cycle repeated. The cells were analyzed for Th2 differentiation and CCR4 expression before use in experiments.

### In Vitro Assay to Measure Antagonist Activity of Molecules

Chemotaxis assay was performed by measuring the ability of molecules to inhibit cellular migration through a 5 µm pore polycarbonate filter in 24-well transwell chambers (Costar, Cambridge, MA). Chemokines (R&D systems) were placed in lower chambers in a 600 µl volume and cells were placed in upper chambers in a 100 µl volume. After 2 h incubation at 37°C, cells in the lower chamber were recovered and counted using a FACSCalibur (Becton Dickinson, Mountain View, CA) [34]. Preliminary chemokine titration experiments established optimal doses for chemotaxis: (1) for CCRF-CEM cells, 6 nM CCL22 and 3 nM CCL17 or CXCL12; (2) for Tregs and Th2 cells, 1.2 nM CCL22 or CCL17. To measure the concentrations required to inhibit 50% of cell migration in the chemotaxis assay, graded doses of antagonists were added to a constant concentration of CCL22. To assess CCR4 antagonism, candidate antagonist compounds (10 nM) were mixed directly with chemokines as indicated. Percent inhibition of chemotaxis by CCR4 antagonists was calculated in relation to controls treated with solvent (DMSO) alone as follows: ([no. cells migrated in the presence of DMSO–no. cells migrated in the presence of antagonist]/no. cells migrated in the presence of DMSO)×100.

### Animals and Immunizations

The construction, design and preparation of pFLAG CMV4 mammalian expression vectors (Sigma-Aldrich) expressing *M. tuberculosis* Rv1818c (CMV1818c) and Rv3812 (CMV3812) has been described previously [35]. SP50 was dissolved in DMSO and mixed with each vaccine to give a final concentration of 1 mM compound in 10% DMSO. 6–8 week old Balb-c mice were immunized intramuscularly in the hind leg with 25 µl vaccine/SP50 mix containing 50 µg CMV1818c or CMV3812 DNA three times at weekly intervals. A fourth booster dose of 25 µg of purified recombinant proteins (Rv1818c and Rv3812) expressed in *E.coli* [35] was given subcutaneously with SP50. The experiments were performed as per the rules of the Indian Institute of Science (Bangalore, India).

Human adenovirus type 5 (AdHu5) expressing the 42 kDa region of merozoite surface protein-1 (Ad-MSP-1_42_) from *P. yoelii* was also used [37]. The adjuvant compounds were dissolved in DMSO and were mixed with each vaccine to give a final concentration of 1 mM compound in 10% DMSO. 6–8 week old female BALB/c mice were immunized once intramuscularly in the hind leg with 25 µl of vaccine/compound mix containing 5×10^10^ vp of Ad-MSP-1_42_ per mouse. They were bled 14 days later. Experiments with Ad-Msp-1_42_ were approved by the animal ethical committee of Oxford University and fully complied with the relevant Home Office guidelines.

### T Cell Proliferation Assay

Splenocytes were harvested from mice immunized with mycobacterial antigens (Rv1818c and Rv3812) 72 hrs post last booster. 0.5×10^6^ splenocytes were cultured in presence of 5 µg/ml of either Rv1818c or Rv3812 recombinant proteins for 5 days. Three mice per group were tested individually. The T cellular proliferative response was measured by thymidine incorporation assay following a 16 h pulse with 1 µCi of [^3^H]thymidine. Radioactive incorporation was measured by standard liquid scintillation counting and results expressed as counts per minute (cpm, mean±SD of triplicate values). The data are presented as stimulation index (SI, Mean cpm of the antigen stimulated wells/Mean cpm of control.

### ELISA

Serum was collected two weeks after Ad-MSP-1_42_ vaccination and analysed by indirect ELISA as previously described using recombinant GST-MSP-1_19_ or GST control followed by alkaline phosphatase-conjugated anti-mouse whole IgG (Sigma) [60]. Endpoint titres were taken as the x-axis intercept of the dilution curve at an absorbance value 3 x standard deviations greater than the OD_405_ for naïve mouse serum (typical cut off OD_405_ for positive sera = 0.15).
